# Exploring the celecoxib–cervical cancer relationship using *in vitro*, network pharmacology, and Mendelian randomization approaches

**DOI:** 10.3389/fmed.2026.1777235

**Published:** 2026-05-19

**Authors:** Tongfei Wang, Xiaohan Zheng, Bing Chen, Ruiying Xiao, Xinyan Peng, Dezhao Chen

**Affiliations:** 1 Department of Gynecology and Obstetrics, Fujian Medical University Union Hospital, Fuzhou, Fujian, China; 2 Key Laboratory of Nanomedical Technology (Education Department of Fujian Province), School of Pharmacy, Fujian Medical University, Fuzhou, Fujian, China; 3 School of Basic Medical Sciences, Fujian Medical University, Fuzhou, Fujian, China

**Keywords:** celecoxib, cervical cancer, immune cells, Mendelian randomization, network pharmacology

## Abstract

**Objective:**

To clarify the potential anticancer role of celecoxib and its immune-related mechanisms.

**Material and method:**

We confirmed the antitumor effects of celecoxib using Cell Counting Kit-8, wound-healing, colony-formation, and transwell assays. Network pharmacology and enrichment analyses were performed to identify pathways linking celecoxib to cervical cancer. Based on these results, two-sample and two-step Mendelian randomization (MR) analyses using blood expression quantitative trait loci instruments were conducted to evaluate the causal effect of NEU1 inhibition—the key celecoxib target—on cervical cancer risk and to assess immune cell mediation.

**Results:**

Celecoxib significantly inhibited cervical cancer cell proliferation, migration, colony formation, and invasion. Network pharmacology and enrichment analyses consistently highlighted T-cell–related immune pathways. MR revealed that genetically proxied NEU1 inhibition reduced cervical cancer risk (*OR* = 0.736, 95% CI: 0.566–0.958), with CD25 on CD45RA^+^ CD4^+^ non-regulatory T cells mediating 28.458% of the total effect.

**Conclusion:**

By integrating genetic causal inference, *in vitro* experiments, and network pharmacology, our study systematically reveals that celecoxib may exert therapeutic effects by targeting against cervical cancer by targeting NEU1 and modulating CD25 on CD45RA^+^ CD4^+^ non-regulatory T cell-related immune pathways. This finding highlights both the novelty and the translational potential of this approach.

## 1 Introduction

Cervical cancer ranks as the fourth most common cancer and the leading cause of cancer-related deaths among women, posing a significant health risk to women (1). In 2022, there were an estimated 660,000 new cases of cervical cancer and approximately 350,000 new deaths worldwide (1). In the United States, the numbers of new cervical cancer cases is projected to be approximately 13,490, with approximately 4,200 deaths expected in 2026 (2). Recently, the introduction of cervical cancer vaccines and screening programs has led to a decline in its global incidence. However, according to the global cancer burden data in 2020, cervical cancer remains one of the malignant tumors with high incidence and mortality rates among women, with approximately 84% of new cases and 88% of related deaths occurring in low- and middle-income countries (3). Although the promotion of vaccination and screening programs has reduced the incidence rate in some regions, cervical cancer remains a significant public health issue in resource-limited areas.

The estimated 5-year survival rate for patients with early-stage cervical cancer exceeds 90%, whereas the curative treatment options for advanced-stage disease are limited (4, 5). The prognosis for advanced cervical cancer is poor because treatment options are limited to chemotherapy or radiotherapy. The median progression-free survival (PFS) ranges from 3 to 6 months, while the median overall survival is 16.8 months (5, 6). Currently, potential improvements in quality of life offered by new therapies remain hindered by drug-related toxicity. In the United States, survival has not significantly improved for cervical cancer patients since the 1970s, demonstrating the urgent need to enhance current treatment approaches (7).

The tumor microenvironment (TME) is recognized to directly influence the therapeutic efficacy and patient outcomes. Cervical cancer is characterized as an immune-infiltrated but immunosuppressive cancer type (8). Single-cell analysis reveals altered immune cell proportions and phenotypes during tumor progression. Populations of innate and adaptive immune cells, including B cells, T cells, regulatory T cells (Tregs), monocytes, neutrophils, and M2-like macrophages, increase, whereas specific subsets such as CD4+ and CD8+ T cells decrease. Cao G et al. reported that T and NK cells were enriched in tumors and subsequently transition to exhausted phenotypes (8). Loss of the CD45RA marker on activated naïve T cells is linked to worse outcomes, hindering the immune response against tumors (9).

Anti-inflammatory drugs such as celecoxib are under investigation for cancer treatment. Celecoxib, a COX-2 inhibitor, induces apoptosis in various tumors, including cervical cancer. Furthermore, it reduces COX-2 expression and markers of proliferation and neoangiogenesis in cervical cancer models, highlighting its potential as a dual strategy for both chemoprevention and treatment (10–13). It may also radiosensitize cervical cancer cells (14). Celecoxib alleviates T-cell and NK-cell exhaustion in hepatocellular carcinoma (15), reduces Treg cell populations (16, 17), and enhances antitumor function of CD8+ T cells (15, 18). Studies on combination therapy with PD-1 inhibitors indicate that celecoxib can facilitate the restoration of a dysregulated tumor immune microenvironment (17). In colon tumors, celecoxib exerts a favorable impact on tumor-infiltrating lymphocytes, thereby contributing to growth control (19).

However, the immunoregulatory mechanisms of celecoxib in cervical cancer have not been fully elucidated. Existing studies suggest that celecoxib modulates T cell activation and the immune microenvironment, yet direct evidence regarding whether these effects are mediated by specific immune cell populations is lacking. This study aims to systematically explore the underlying immune-mediated mechanisms of celecoxib and its target genes in cervical cancer by integrating *in vitro* experiments, network pharmacological analysis, and Mendelian randomization, thereby filling the current gap in evidence concerning immune-related mechanisms.

## 2 Materials and methods

### 2.1 Cell culture and reagents

Human cervical cancer cell lines HeLa and SiHa (both Serial No. SCSP-504), obtained from the Cell Bank of the Chinese Academy of Sciences (Shanghai, China), were maintained in Dulbecco's Modified Eagle Medium (DMEM) supplemented with 10% fetal bovine serum and 1% penicillin–streptomycin at 37 °C in a humidified 5% CO_2_ incubator. Celecoxib was dissolved in DMSO to prepare stock solutions, which were then diluted to working concentrations immediately prior to use.

### 2.2 Cell viability assay (CCK-8)

Cell viability was assessed using the Cell Counting Kit-8 (CCK-8). Cells were seeded into 96-well plates, treated with gradient concentrations of celecoxib, and incubated for 24–48 h. Absorbance at 450 nm was measured, and inhibitory effects were calculated relative to control wells.

### 2.3 Wound-healing assay

Cells were grown to confluence in 6-well plates. A sterile pipette tip was used to create a linear scratch, followed by treatment with celecoxib in serum-free medium. Wound closure was photographed at 0 and 24 h, and migration rates were quantified using ImageJ.

### 2.4 Colony-formation assay

Cells were seeded into 6-well plates at low density and exposed to celecoxib. After 10–14 days of incubation, cell colonies were fixed with paraformaldehyde, stained with crystal violet, and counted manually. Colony-formation efficiency was calculated relative to controls.

### 2.5 Transwell invasion assay

Cell invasion was evalated via Matrigel-coated Transwell chambers. Cells were seeded in the upper chamber in serum-free medium supplemented with celecoxib, whereas the lower chamber was filled with medium containing 10% serum. Following a 24-h incubation, invaded cells were fixed, stained, and quantified via microscope.

Statistical differences between the DMSO control and various celecoxib concentration groups were analyzed using one-way ANOVA.

### 2.6 Network pharmacology analysis

To analyze how celecoxib acts on cervical cancer cells, the potential targets of celecoxib were retrieved from the following databases: SwissTargetPrediction, and PubChem (accessed in December 2025). For, PubChem, only human (Homo sapiens) targets were included. For SwissTargetPrediction, targets with probability ≥0.1 were retained. Duplicate entries were removed after merging results from different databases. Cervical cancer related genes were collected from GeneCards, and OMIM (accessed in December 2025). In GeneCards, genes with a relevance score ≥10 were selected. All retrieved genes were standardized using official gene symbols. The overlapping genes between celecoxib targets and cervical cancer–related genes were identified using Venn analysis. These intersecting genes were considered potential therapeutic targets.

Gene Ontology (GO) and Kyoto Encyclopedia of Genes and Genomes (KEGG) pathway enrichment analyses were performed using the R package cluster Profiler (version 4.8.1). A *p*-value < 0.05 and false discovery rate (FDR) < 0.05 were considered statistically significant.

The protein–protein interaction (PPI) network was constructed using the STRING database (version 11.5), with the species restricted to Homo sapiens and a minimum required interaction score ≥0.7 (high confidence). The network was visualized and analyzed using Cytoscape (version 3.9.1). Key hub genes were identified using the CytoHubba plugin based on degree centrality.

### 2.7 MR analysis study design

**Figure 1:**
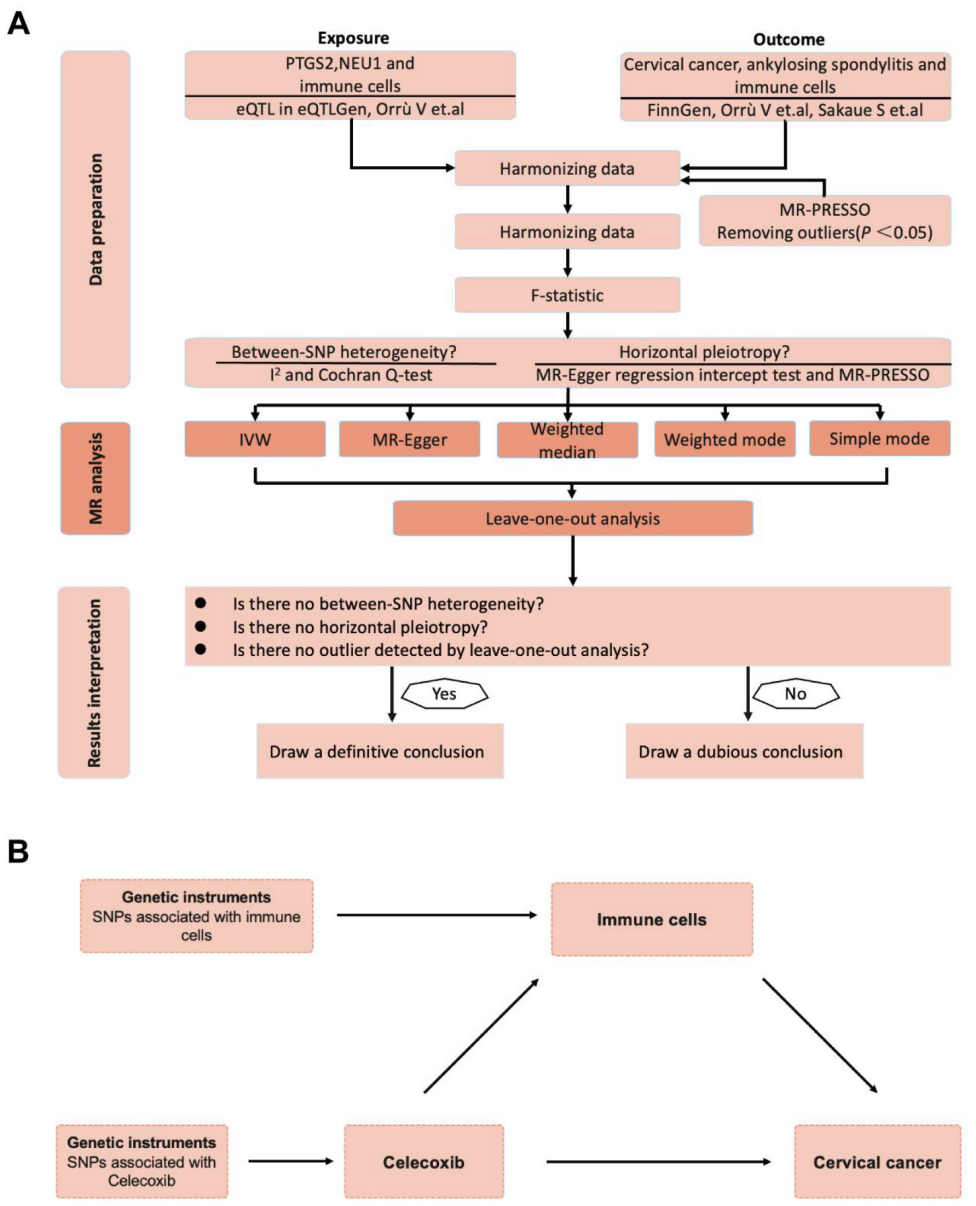
Overview of the study design. **(A)** The flowchart of Mendelian randomization analysis. **(B)** The framework of the two-step Mendelian randomization. PTGS2, prostaglandin-endoperoxide synthase 2; NEU1, neuraminidase 1; eQTL, expression quantitative trait loci; SNP: single nucleotide polymorphism; IVW, inverse variance weighted; MR, Mendelian randomization.

This study employed a two-sample, two-step MR analysis to estimate the causal effects linking celecoxib target genes, immune cells, and cervical cancer (Figure 1). Sensitivity analyses, including the evaluation of pleiotropy and heterogeneity, were conducted to assess the robustness of the identified MR associations. Four key assumptions must be met to validate potential causal effects (20): (1) genetic variants are significantly associated with the exposure (“relevance”), (2) they must be independent of confounding factors related to the exposure (“independence”), (3) they must not directly correlate with outcomes but only influence them through the exposure (“exclusion”), and (4) they must fall within the cis-acting region of the target gene. Furthermore, this study also adhered to the requirements of STROBE-MR (21). Benjamini–Hochberg false discovery rate (FDR) correction was applied to all Mendelian randomization analyses and functional enrichment analyses involving multiple comparisons. Adjusted *P*-values (FDR-adjusted *P* < 0.05) were considered statistically significant.

### 2.8 Data sources

The celecoxib target genes prostaglandin-endoperoxide synthase 2 (PTGS2, also known as COX-2) and neuraminidase 1 (NEU1), which have pharmacological effects, were obtained from the DrugBank database (https://go.drugbank.com/drugs/DB00482). We retrieved publicly available genome-wide association study (GWAS) summary data for expression quantitative trait loci (eQTLs) associated with celecoxib target genes (eQTL-a-ENSG00000073756 and eQTL-a-ENSG00000204386), alongside data regarding 731 immune cells (22) and cervical cancer (ebi-a-GCST90018817) from the IEU OpenGWAS project database (https://gwas.mrcieu.ac.uk/; Table 1). To validate our selection of instruments for celecoxib target genes, we conducted a positive control analysis using ankylosing spondylitis (AS). As a COX-2 inhibitor, celecoxib is widely utilized in treating inflammatory diseases, while NEU1 is known to promote inflammatory responses and autoimmune processes. AS is a prototypical chronic inflammatory disease; its pathological mechanisms overlap with the celecoxib's mechanism of action, thereby establishing it as a robust positive control for validiting the genetic instruments. The summary data for AS were from FinnGen R10 database (https://www.finngen.fi/fi), which accumulated association summary statistics from GWAS in 410,618 sample size and 1,381 cases (Table 1). We extracted European ancestry-specific GWAS data for this study. Data derived from GWAS and eQTLs were obtained solely from open-access repositories that have secured ethical approval.

**Table 1:** Brief information of eQTL^a^ and GWAS^a^ database in Mendelian randomization.

Data source	Phenotype	Sample size	Cases	Population	Adjustment
eqtl-a-ENSG00000073756	PTGS2^a^	31,470	—	European	Males and females
eqtl-a-ENSG00000204386	NEU1^a^	14,263	—	European	Males and females
ebi-a-GCST90018817	Cervical cancer	239,158	909	European	—
Finngen_R10_M13_ANKYLOSPON_STRICT	Ankylosing spondylitis, strict definition	410,618	1,381	European	Males and females
IEU open GWAS project	Immune cells	—	—	European	—

^a^eQTL, expression quantitative trait loci; GWAS, genome-wide association study; PTGS2, prostaglandin-endoperoxide synthase 2; NEU1, neuraminidase 1.

### 2.9 Genetic instrument selection

#### 2.9.1 Genetic instruments for celecoxib target genes

We selected eQTLs within ±300 kb of the cis-acting regions of celecoxib target genes as genetic instruments, specifically single nucleotide polymorphisms (SNPs). We applied a screening criterion of *P* < 1 × 10^−5^ to ensure a stronger correlation between the selected SNPs and the target phenotype. We further grouped the SNPs based on a linkage disequilibrium (LD) threshold of *r*^2^ < 0.30, a physical distance of 100 kb, and a minor allele frequency (MAF) greater than 0.05. The SNPs related to confounding factors and outcomes were excluded by using LDtrait (https://ldlink.nih.gov/?tab=ldtrait). Then, extract the relevant SNPs selected above from the GWAS summary data of the outcome variables (cervical cancer, immune cells, and AS), and exclude SNPs directly associated with the outcome variables with a significance threshold of *P* < 1 × 10^−5^, as well as those with palindromic alleles and MAF > 0.42. Strength of the genetic instruments were evaluated by *F* statistics, where *F* statistics >10 was indicative of adequate strength. The outlier SNPs were extracted using the Mendelian Randomization Pleiotropy RESidual Sum and Outlier (MR-PRESSO).

#### 2.9.2 Genetic instruments for immune cells

We selected SNPs for each immune cell that achieved genome-wide significance (*P* < 1 × 10^−5^) and were independent of each other (*r*^2^ < 0.001) within a physical distance of 10,000 kb. The SNPs related to confounding factors and outcomes were excluded by using LDtrait. The relevant SNPs were extracted from the GWAS summary data for the outcome (cervical cancer). We assessed the strength of the genetic instruments using *F*-statistics, where an *F*-statistic >10 was considered indicative of robust instrument strength. We removed SNPs directly associated with outcome variables (*P* < 1 × 10^−5^), along with those having palindromic alleles and MAF greater than 0.42. Outlier SNPs were removed using the MR-PRESSO method.

### 2.10 Statistical analyses

#### 2.10.1 MR analysis to estimate the effects of celecoxib target genes on cervical cancer and AS

We employed two-sample univariable Mendelian Randomization (UVMR) to estimate the effects of celecoxib-targeted genes on cervical cancer and AS. The analysis employed the Inverse variance weighted (IVW), MR-Egger, weighted median, weighted mode, and simple mode methods. The IVW method served as the primary analytical approach. MR-Egger regression can identify and correct for pleiotropy, although its estimation accuracy is quite low (23). The weighted median method provides a consistent estimate of the causal relationship between exposure and outcome when at least 50% of the genetic variants are valid (24). The simple mode is a model-based approach capable of providing robustness against pleiotropy. For mode assessment, the weighted mode is sensitive to the hard throughput collection (25).

#### 2.10.2 Mediation MR analysis linking celecoxib target genes with cervical cancer via immune cells

The mediation analysis in this study was conducted using a two-step MR framework, based on existing methodological literature (26). When reporting the mediation proportion, we only include results where the primary causal pathway direction is consistent and statistically significant to ensure the validity of the inference. A two-step MR was conducted to estimate the mediating role of immune cells in the association between celecoxib target genes and cervical cancer (Figure 1B). First, we utilized univariable Mendelian Randomization (UVMR) to estimate the effect of celecoxib target genes on immune cells (β1). Second, we estimated the effect of immune cells on cervical cancer using UVMR (β2). We calculated the mediation proportions for each immune cell in the association between celecoxib target genes and cervical cancer using the formula: (β1 × β2)/β3. Here, β3 represents the total effect of celecoxib target genes on cervical cancer, derived from our primary analysis. The mediation effect can be reported when the directions of the total effect, direct effect, and indirect effect are aligned, and the mediation proportion exceeds 5%.

#### 2.10.3 Sensitivity analysis

In this study, the heterogeneity of MR analysis was assessed using IVW and MR-Egger. We used Cochran's *Q* test and *I*^2^ statistics to evaluate the heterogeneity of SNPs. For the Cochran's *Q* test, a *P*-value of < 0.05 represented significant heterogeneity. For *I*^2^ statistics, an *I*^2^ value exceeding 50% indicated substantial heterogeneity. We performed “leave-one-out” sensitivity analyses by iteratively omitting each SNP to determine whether any single variant was driving the observed association. Moreover, the MR-Egger intercept analysis and MR-PRESSO were employed to assess horizontal pleiotropy. The absence of a significant difference between the MR-Egger regression intercept and zero (*P* > 0.05), combined with a non-significant MR-PRESSO global test (*P* > 0.05), suggested no evidence of horizontal pleiotropy.

All MR analyses were conducted with the package “TwoSampleMR” in R software (version 4.1.0). A *P*-value < 0.05 was considered statistically significant.

## 3 Results

### 3.1 Celecoxib suppresses proliferation, migration, invasion, and colony-forming ability of cervical cancer cells

**Figure 2:**
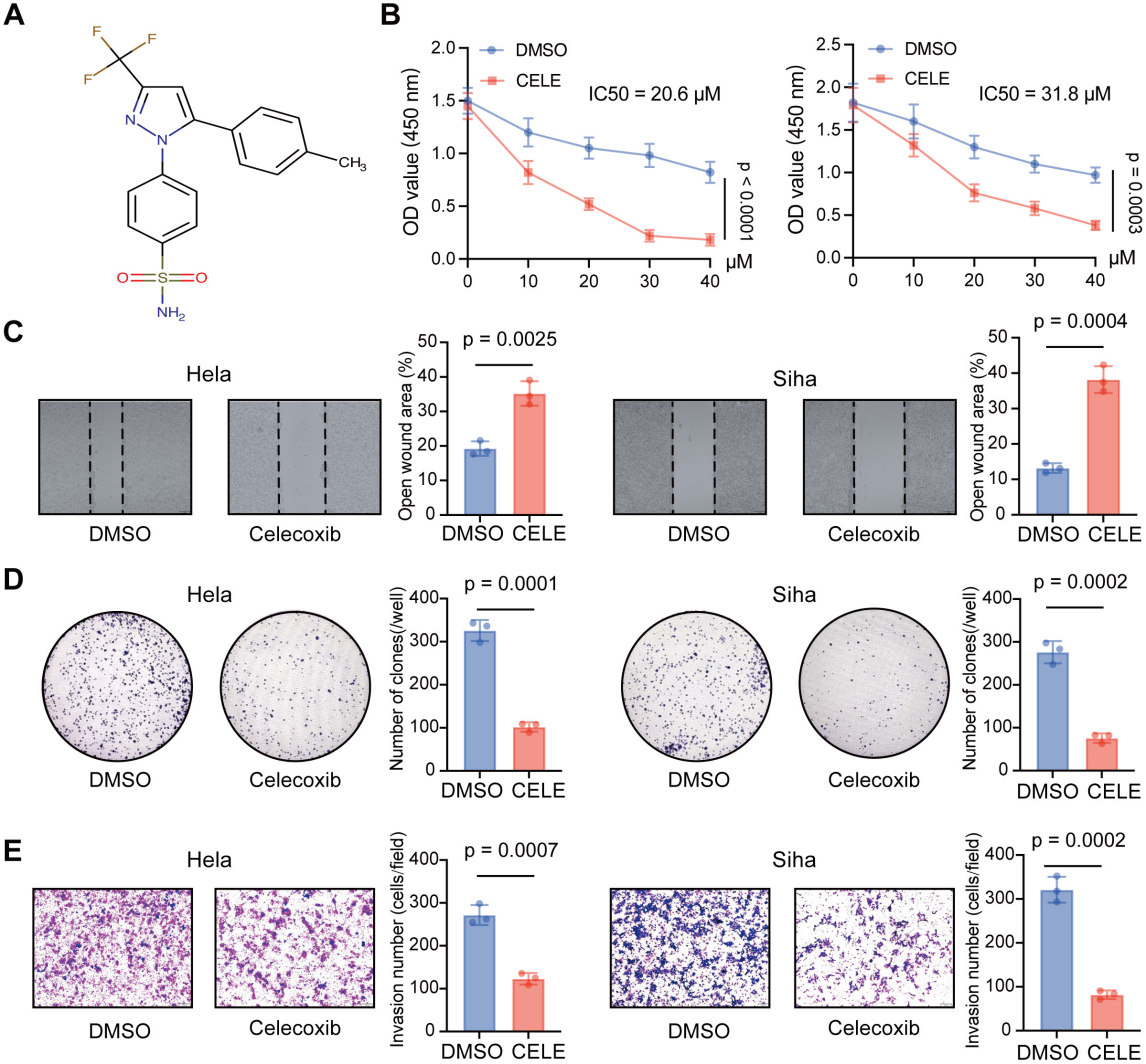
Celecoxib suppresses cervical cancer cell viability, migration, clonogenicity, and invasion. **(A)** Chemical structure of celecoxib. **(B)** CCK-8 assays showing celecoxib-induced reduction in cell viability in HeLa and SiHa cells. **(C)** Wound-healing assays demonstrating impaired migratory ability of HeLa and SiHa cells following celecoxib treatment. **(D)** Colony-formation assays showing decreased clonogenic growth after celecoxib exposure. **(E)** Transwell invasion assays indicating reduced invasive capacity of HeLa and SiHa cells upon celecoxib treatment. In our experiments, celecoxib was dissolved in DMSO, and the control group received an equal volume of DMSO (vehicle control). All experiments were performed independently at least three times (*n* = 3). Data are presented as mean ± standard deviation (SD). For CCK-8 cell viability assays involving different drug concentrations and treatment conditions, statistical differences were analyzed using two-way analysis of variance (ANOVA) followed by Tukey's multiple comparisons test. For comparisons between two groups, an unpaired two-tailed Student's *t*-test was used. *P*-values were calculated accordingly, and *P* < 0.05 was considered statistically significant.

To validate the antitumor effects of celecoxib (Figure 2A), we performed a series of *in vitro* functional assays using HeLa and SiHa cells. CCK-8 assays showed that celecoxib significantly inhibited cell viability in a dose- and time-dependent manner (Figure 2B). Specifically, celecoxib inhibited cell viability in a dose-dependent manner, with IC_50_ values of 20.6 μm and 31.8 μm in HeLa and SiHa cell lines, respectively. Wound-healing assays revealed that celecoxib significantly impeded cell migration compared with controls (Figure 2C). Consistently, colony-formation assays demonstrated a significant decrease in the number and size of colonies following celecoxib treatment, indicating impaired long-term proliferative capacity (Figure 2D). Similarly, Transwell invasion assays showed a substantial decrease in the number of invaded cells after treatment, confirming that celecoxib suppresses the invasive phenotype of cervical cancer cells (Figure 2E). Collectively, these results indicate that celecoxib potently inhibits cervical cancer progression by impairing cell proliferation, colony formation, migration, and invasion.

### 3.2 Network pharmacology analysis identifies immune-related pathways as potential mechanisms underlying celecoxib's effects

**Figure 3:**
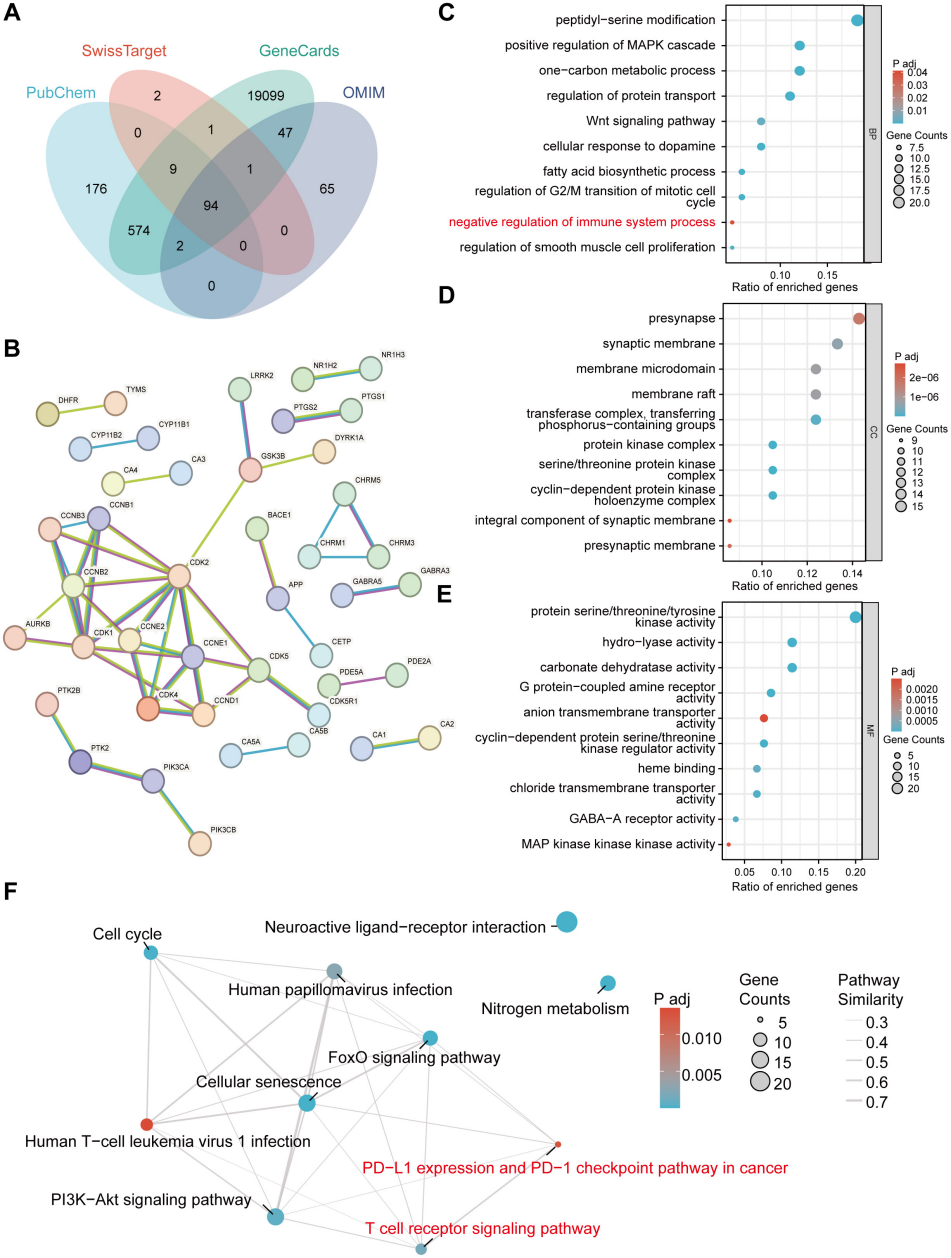
Network pharmacology identifies immune-related pathways potentially involved in celecoxib's effects on cervical cancer. **(A)** Venn diagram showing overlapping genes between cervical cancer–related genes and celecoxib target genes. **(B)** Protein–protein interaction (PPI) network constructed from the intersecting genes. **(C–E)** GO enrichment analysis of the intersecting genes, including Biological Process (BP), Cellular Component (CC), and Molecular Function (MF) categories. **(F)** KEGG pathway enrichment network illustrating the major signaling pathways associated with the intersecting genes.

To explore the molecular mechanisms linking celecoxib to cervical cancer, we performed network pharmacology analyses. Candidate celecoxib targets and cervical cancer–related genes were intersected to obtain a set of overlapping genes (Figure 3A). Next, a protein–protein interaction (PPI) network was constructed using the intersecting genes (Figure 3B). These intersecting genes were subjected to GO and KEGG enrichment analyses. GO analysis revealed significant enrichment in immune-related biological processes, including positive regulation of the MAPK cascade, the Wnt signaling pathway, cellular response to dopamine, the fatty acid biosynthetic process, regulation of G2/M transition of the mitotic cell cycle, and negative regulation of the immune system process (Figures 3C–E). KEGG pathway analysis consistently showed that these genes were predominantly enriched in immune pathways, particularly those related to T-cell function, such as the T-cell receptor signaling pathway, Human T–cell leukemia virus 1 infection, and the PD–L1 expression and PD−1 checkpoint pathway in cancer (Figure 3F). These results indicate that the celecoxib–cervical cancer axis is closely associated with immune regulation. The repeated enrichment of T-cell–related pathways suggests that modulation of T-cell activity may be a central mechanism through which celecoxib exerts its therapeutic or antitumor effects.

### 3.3 Causal effects of celecoxib target genes on cervical cancer

Given that our *in vitro* assays demonstrated clear antitumor effects of celecoxib and that network pharmacology analyses suggested immune- and T-cell–related pathways as potential mechanisms, we next applied Mendelian randomization (MR) to evaluate whether celecoxib's molecular targets exert causal effects on cervical cancer.

We employed a two-sample, two-step MR analysis to estimate the causal effects among celecoxib target genes, immune cells, and cervical cancer (Figure 1). We first evaluated celecoxib's two primary DrugBank targets, PTGS2 and NEU1, for the construction of genetic instruments. We identified 17 independent cis-eQTL SNPs for PTGS2 and 13 for NEU1, all with *F* statistics >20, indicating adequate instrument strength (Supplementary Table S1). These SNPs were then used to estimate the causal associations between target inhibition and cervical cancer (Table 2). In the MR analysis, genetically proxied NEU1 inhibition was associated with a significantly lower risk of cervical cancer (*OR* = 0.736, 95% CI: 0.566–0.958, *P* = 0.023). Sensitivity analyses showed no evidence of horizontal pleiotropy (MR-Egger intercept *P* = 0.407; MR-PRESSO *P* = 0.582) and no heterogeneity (*I*^2^ = 0%, *Q* = 10.430, *P* = 0.578), thereby supporting the robustness of these finding. In contrast, no statistically significant causal association was observed for PTGS2 (*P* = 0.994; Table 2).

**Table 2:** Mendelian randomization estimates of the effect of celecoxib target genes on cervical cancer and ankylosing spondylitis.

Outcome	Exposure	Method	MR^a^		Heterogeneity			Horizontal pleiotropy		
			Drug_OR (95%CI)	*P*-value	*I*^2^ (%)	Cochran's *Q*	*P*-value	Egger intercept	*P*-value	MR-PRESSO *P*-value
Cervical cancer	PTGS2^a^ inhibitor	Inverse variance weighted	0.999 (0.838–1.192)	0.994	0	13.294	0.651	—	—	0.665
		MR Egger	0.853 (0.523–1.391)	0.533	0	12.830	0.615	−0.021	0.506	0.665
		Simple mode	0.845 (0.564–1.266)	0.425	—	—	—	—	—	0.665
		Weighted median	0.906 (0.708–1.160)	0.436	—	—	—	—	—	0.665
		Weighted mode	0.869 (0.636–1.188)	0.392	—	—	—	—	—	0.665
	NEU1^a^ inhibitor	Inverse variance weighted	0.736 (0.566–0.958)	0.023	0	10.430	0.578	-	-	0.582
		MR Egger	1.072 (0.438–2.621)	0.882	0	9.686	0.559	0.039	0.407	0.582
		Simple mode	0.800 (0.444–1.444)	0.474	—	—	—	—	—	0.582
		Weighted median	0.761 (0.531–1.090)	0.137	—	—	—	—	—	0.582
		Weighted mode	0.821 (0.463–1.455)	0.512	—	—	—	—	—	0.582
Ankylosing spondylitis	NEU1^a^ inhibitor	Inverse variance weighted	0.066 (0.034–0.126)	< 0.001	0	0.288	0.998	—	—	0.999
		MR Egger	0.086 (0.016–0.467)	0.047	0	0.167	0.997	0.033	0.746	0.999
		Simple mode	0.062 (0.020–0.194)	0.005	—	—	—	—	—	0.999
		Weighted median	0.066 (0.027–0.161)	< 0.001	—	—	—	—	—	0.999
		Weighted mode	0.069 (0.024–0.192)	0.004	—	—	—	—	—	0.999

^a^PTGS2: prostaglandin-endoperoxide synthase 2; NEU1: neuraminidase 1; MR: Mendelian randomization.

Overall, these results demonstrate that only NEU1 exhibited a genetic association whose direction was consistent with celecoxib exposure and exhibited a protective causal effect on cervical cancer, indicating that NEU1—not PTGS2—may constitute the key mechanistic link underlying celecoxib's antitumor activity.

### 3.4 Causal effects of NEU1 inhibition on ankylosing spondylitis (as positive control)

Given that only NEU1 showed a causal association with cervical cancer in our primary MR analysis (Table 2), we next evaluated ankylosing spondylitis (AS) as a positive control outcome to validate the reliability of the NEU1 instruments. From the cis-eQTL dataset, 6 independent SNPs for NEU1 were available for the AS analysis. MR estimates indicated that genetically proxied NEU1 inhibition markedly reduced AS risk (*OR* = 0.066, 95%CI: 0.034–0.126, *P* < 0.001; Table 2). Sensitivity analyses indicated no evidence of horizontal pleiotropy (Egger intercept *P* = 0.746; MR-PRESSO *P* = 0.999) and no heterogeneity (*I*^2^ = 0%, *Q* = 0.288, *P* = 0.998), with leave-one-out analysis confirming stability of the results. This positive-control association further supports the validity of the NEU1 genetic instruments and strengthens the causal inference observed in the cervical cancer analysis.

### 3.5 Causal effects of NEU1 inhibitor, immune cells, and cervical cancer

**Figure 4:**
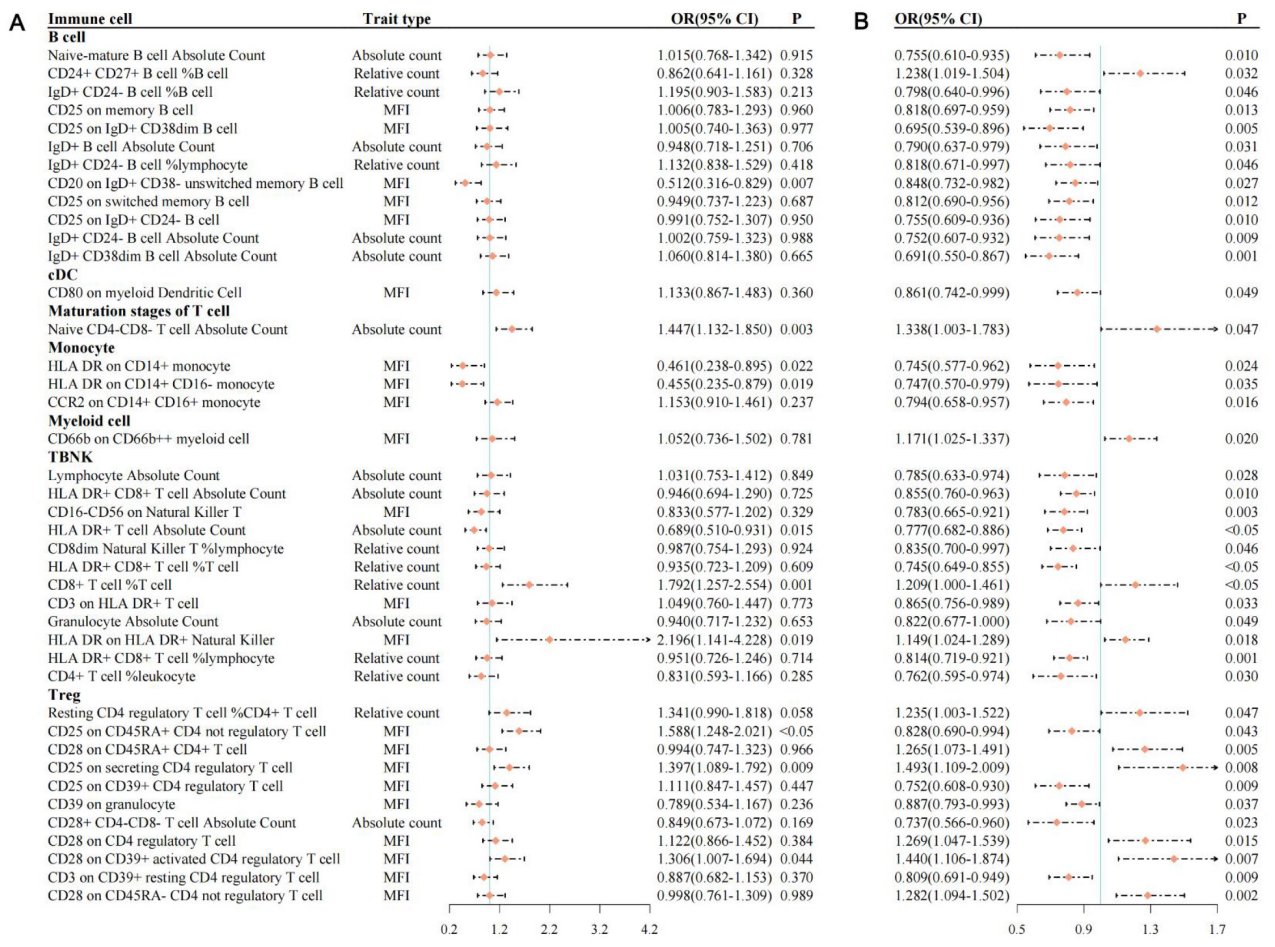
The forest plot of showing the effects of celecoxib on immune cells and the effects of immune cells on cervical cancer. **(A)** The effects of celecoxib on immune cells. **(B)** The effects of immune cells on cervical cancer. *OR*: odds ratio

Given the MR evidence supporting NEU1—rather than PTGS2—as the celecoxib-relevant target in cervical cancer, we next employed a two-step MR framework to evaluate whether NEU1 inhibition may influence cervical cancer through immune-cell pathways. Using the NEU1 cis-eQTL dataset, we identified 9,768 SNPs associated with immune-cell traits (Supplementary Table S2); of these, 7,200 immune-cell–related SNPs were subsequently mapped to cervical cancer outcomes (Supplementary Table S3). Across 731 immune-cell phenotypes, NEU1 inhibition exerted significant effects on 162 immune-cell traits (Supplementary Table S4; Figure 4A). Notably, NEU1 inhibition increased CD45RA^+^ CD28– CD8– T-cell absolute counts and decreased CD8 expression on CD28^+^ CD45RA^+^ CD8^+^ T cells (*OR* = 5.08 × 10^2^^2^, *P* = 0.010; *OR* = 0.380, *P* < 0.001), respectively, with no evidence of heterogeneity (Cochran's *Q P* > 0.5) or horizontal pleiotropy (Egger intercept *P* > 0.80; MR-PRESSO *P* > 0.50).

We then assessed the influence of these 731 immune-cell traits influenced cervical cancer risk and identified 41 immune-cell phenotypes significantly associated with the disease (Supplementary Table S5; Figure 4B). Higher absolute counts of IgD^+^ CD38^∧^dim B cells were associated with lower cervical cancer risk (*OR* = 0.691, *P* = 0.001), whereas elevated CD25 expression on secreting CD4 regulatory T cells was associated with increased susceptibility (*OR* = 1.493, *P* = 0.008). Sensitivity analyses showed no evidence of heterogeneity (Cochran's *Q P* > 0.65) or horizontal pleiotropy (Egger intercept *P* > 0.58; MR-PRESSO *P* > 0.70).

Collectively, these results indicate that NEU1 inhibition has broad and specific immunomodulatory effects, while several of the affected immune cell subsets also influence cervical cancer risk. This provides mechanistic support for a potential causal pathway wherein NEU1 drives immune-cell alterations that subsequently influence cervical cancer.

### 3.6 Mediating effects of immune cells on the association between NEU1 inhibition and cervical cancer

**Figure 5:**
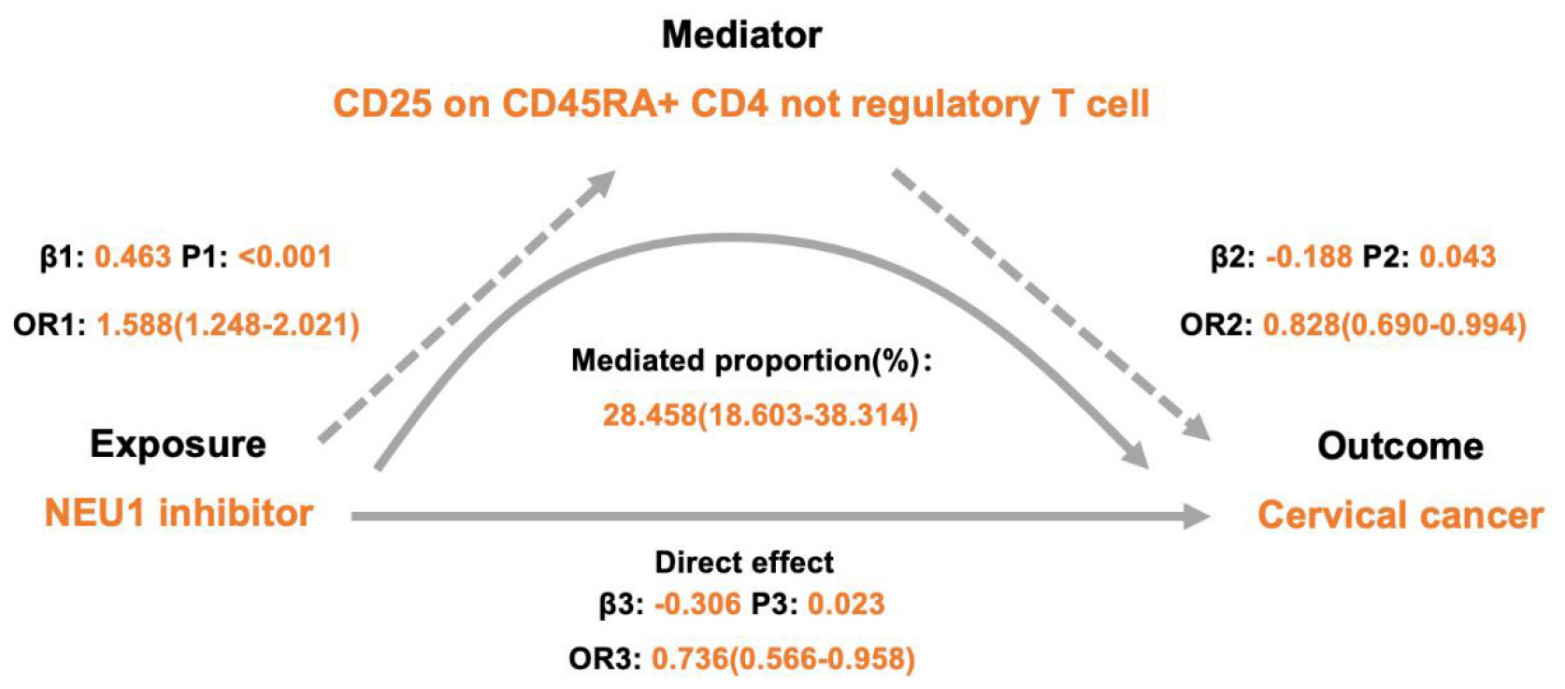
Mediation analysis of the effect of celecoxib on cervical cancer via immune cells. NEU1, neuraminidase 1; *OR*, odds ratio.

Based on the two-step MR results, we evaluated 10 immune cell traits as potential mediators, the detailed estimates of which are presented in Table 3. Among them, CD25 on CD45RA^+^ CD4^+^ non-regulatory T cells was identified as the sole significant mediator, accounting for 28.458% of the association between NEU1 inhibitor levels and cervical cancer risk (Table 3, Figure 5). Genetically increased NEU1 inhibition was associated with a lower cervical cancer risk (effect = −0.306; *OR* = 0.736, 95% CI: 0.566–0.958, *P* = 0.023), and was concurrently associated with elevated CD25 expression on CD45RA^+^ CD4^+^ non-regulatory T cells (effect = 0.463). Elevated levels of this immune trait were in turn associated with reduced cervical cancer risk (effect = −0.188; Figure 5).

**Table 3:** Mediation effects of celecoxib on cervical cancer via immune cells.

Mediation	Mediating effect	Direct effect	Total effect	Proportion mediated (%)
Naïve CD4-CD8- T cell absolute count	0.107	−0.414	−0.306	−35.076 (−48.374 to −21.777)
CD8+ T cell %T cell	0.111	−0.417	−0.306	−36.084 (−49.453 to −22.715)
HLA DR+ T cell absolute count	0.094	−0.400	−0.306	−30.661 (−39.894 to −21.429)
CD20 on IgD+ CD38- unswitched memory B cell	0.111	−0.417	−0.306	−36.154 (−49.327 to −22.980)
CD28 on CD39+ activated CD4 regulatory T cell	0.097	−0.404	−0.306	−31.783 (−44.084 to −19.481)
CD25 on CD45RA+ CD4 non-regulatory T cell	−0.087	−0.219	−0.306	28.458 (18.603 to −38.314)
CD25 on secreting CD4 regulatory T cell	0.134	−0.440	−0.306	−43.681 (−58.259 to −29.102)
HLA DR on CD14+ CD16- monocyte	0.229	−0.536	−0.306	−74.870 (−104.935 to −44.804)
HLA DR on CD14+ monocyte	0.228	−0.534	−0.306	−74.325 (−103.432 to −45.219)
HLA DR on HLA DR+ natural killer	0.109	−0.415	−0.306	−35.593 (−48.985 to −22.201)

The two-step MR analysis supported this mediation pattern. In step one, NEU1 inhibition was associated with increased expression of CD25 on CD45RA^+^ CD4^+^ non-regulatory T cells (*OR* = 1.588, 95% CI: 1.248–2.021, *P* < 0.001). In the second step, this immune trait demonstrated a protective association against cervical cancer (*OR* = 0.828, 95% CI: 0.690–0.994, *P* = 0.043; Supplementary Tables S4, S5, Figure 5). Sensitivity analyses revealed no heterogeneity (*I*^2^ = 0%; Cochran's *Q* = 9.523, *P* = 0.732; 7.249, *P* = 0.611) and no horizontal pleiotropy (Egger intercept = 0.005, *P* = 0.907; 0.010, *P* = 0.852; MR-PRESSO *P* = 0.764, 0.676). All instruments variables demonstrated sufficient strength (*F* > 19).

We calculated the *F*-statistics for each major instrumental variable, including the target genes of celecoxib (PTGS2, NEU1) and their corresponding key SNPs, and have included the detailed results in Supplementary Table S6.

## 4 Discussion

In this study, we integrated *in vitro* experiments, network pharmacology, and MR to comprehensively evaluate the therapeutic potential of celecoxib in cervical cancer. Our *in vitro* assays demonstrated the antitumor effect of celecoxib on cervical cancer cells, and network pharmacology further revealed that the overlapping genes were predominantly enriched in immune-related pathways—particularly T-cell–associated signaling. Guided by these findings, we conducted MR analyses to clarify causal relationships and identified NEU1, rather than PTGS2, as the celecoxib target gene with a genetic pattern consistent with drug exposure. Genetically proxied NEU1 inhibition was associated with a reduced risk of cervical cancer and was subsequently validated using AS as a positive control (cervical cancer: *OR* = 0.736, 95% CI: 0.566–0.958; AS: *OR* = 0.066, 95% CI: 0.031–0.126). Furthermore, the key findings of this study (NEU1 inhibition–CD25 on CD45RA^+^ CD4^+^ non-regulatory T cells – cervical cancer) remained significant after multiple comparison correction, indicating a certain degree of robustness in the related mediating effects. Mediation analyses indicated that CD25 on CD45RA^+^ CD4^+^ non-regulatory T cells accounted for 28.5% of the protective effect of NEU1 inhibition on cervical cancer, highlighting an immune-cell–mediated pathway underlying celecoxib's potential benefit.

Celecoxib, a selective inhibitor of COX-2, has been in use for more than 20 years. Due to its anti-proliferative properties, celecoxib has been an attractive drug in recent years. Increased COX-2 expression was reported to be associated with chemoresistance and poor prognosis of cervical cancer (27). Inhibiting COX-2 can enhance the therapeutic potential for cervical cancer (27, 28). Numerous studies have shown that inhibiting COX-2 expression with celecoxib promotes apoptosis and significantly reduces cancer cell proliferation (12, 29–31). Additionally, celecoxib demonstrated dose-dependent efficacy in animal models without showing toxicity (32). A study has found that celecoxib enhances the radiosensitivity of the human cervical cancer HeLa cell line by reducing the expression of COX-2 and vascular endothelial growth factor C (14). Conversely, celecoxib has also been shown to inhibit the proliferation of specific cell lines that lack COX-2 expression *in vitro*. Studies have pointed to COX-2-independent mechanisms such as cell cycle arrest, the induction of the mitochondrial pathway of apoptosis, and necrosis (10, 12, 33). Celecoxib was revealed to have high anticancer potential against several metastatic cancer cells such as human cervix, human breast, and melanoma cancer cells (12, 34–36). In the therapy of cervical cancer, celecoxib was found to have a sensitizing effect with other antitumor drugs (such as dichloroacetate, cisplatin, paclitaxel, and doxorubicin) or photodynamic therapy (20, 35, 37). Nowadays, the combination of celecoxib with canonical chemotherapy may represent a promising chemotherapy strategy against cervical cancer growth, because it can selectively block multiple cell processes including inhibition of energy pathways and, in consequence, ATP-dependent processes, without significantly affecting non-cancerous cells (12). Consistent with the aforementioned studies, this study provided strong evidence of the protective effect of celecoxib against cervical cancer by using a set of robust genetic instruments targeting the celecoxib-related gene NEU1 as instrumental variables and a large GWAS for cervical cancer. Nevertheless, a systematic review and meta-analysis revealed that adding celecoxib to standard chemotherapy did not improve overall survival or progression-free survival (PFS) rates in patients with gynecological cancers (38). This result might be due to variations in chemotherapy regimen, different experimental designs, and data acquisition standards, etc. Thus, further well-designed, large-scale, randomized controlled trials (RCT) are needed to investigate the effect of celecoxib on cervical cancer.

NEU1, as one type of mammalian sialidase, is involved in the development of several cancers. Although many previous studies have reported that NEU1 functions as an oncogene and is upregulated in cancer cells, and increasing number of studies show that the role of NEU1 in different tumors is inconsistent. It is reported that NEU1 act as an oncogene in hepatocellular carcinoma, breast cancer, ovarian cancer, and melanoma (39–42). In contrast, NEU1 acts as a tumor suppressor in bladder and colon cancers (43, 44). In pancreatic cancer, NEU1 is believed to promote tumor growth (45). However, in a pancreatic ductal adenocarcinoma cell line model, NEU1 exhibits antitumor effects (46).

Currently, the potential role of NEU1 in cervical cancer remains unclear. The existing body of research on the interrelation between NEU1 and cervical cancer remains scant and limited in scope. A high-resolution proteomics study of the secretome from cervical cell lines confirmed that NEU1 was overexpressed in SiHa and C33A, a finding further validated by the multiple reaction monitoring method (47). Consistent with the aforementioned study, this study provided strong evidence demonstrating the effectiveness of the NEU1 inhibitor in providing a protective effect against cervical cancer. We speculate that this may be attributed to tissue-specific glycosylation backgrounds, differences in the immune microenvironment, and activation patterns of downstream signaling pathways. On the one hand, NEU1 affects cell adhesion, migration, and signal transduction by regulating the desialylation of membrane receptors (such as EGFR, TGF-β, Integrin α5β1) (43). On the other hand, in the immune-enriched but immunosuppressive microenvironment of cervical cancer, it may promote immune escape by altering antigen presentation and T-cell activation thresholds (8). Inhibition of NEU1 may reverse these effects, restore T-cell function, and enhance anti-tumor immune responses. However, Xie N et al. suggested that NEU1 expression was significantly lower in cervical cancer tissues and cells (48). Their study demonstrated that downregulation of NEU1 exacerbated the aggressiveness of cervical cancer and that upregulation of NEU1 suppressed the proliferative, migratory, and invasive abilities of cervical cancer cells (48). Therefore, additional studies are needed to achieve a comprehensive understanding of NE1′s effects on cervical cancer.

The interaction between immune cells and the tumor microenvironment (TME) is widely recognized as a crucial factor in the development and immune evasion of cervical cancer. Studies have shown that most NK cells within the cervical cancer microenvironment are exhausted, and the majority of tumor-infiltrating T cells are also functionally impaired by tumor antigens (49). Furthermore, the CD4+ T cell response is predominantly driven by T helper type 17 (Th17) cells (8), consistent with finding indicating a lack of cell-mediated immunity (T helper type 1 (Th1) response) in cervical cancers (50, 51). In the cervical cancer microenvironment, macrophages polarize from the M1 phenotype to the M2 phenotype (52). Additionally, a reduced abundance of dendritic cells (DCs) coupled with an increased prevalence of regulatory T cells (Tregs) contributes to cervical cancer progression (53).

NEU1 plays a crucial role in the immune system. A study found that NEU1-induced desialylation activates various immune cells, such as macrophages, neutrophils, and monocytes (54). Activated T lymphocytes have been shown to increase the production of inflammatory cytokines such as TNF-α and IFN-γ mediated by NEU1 or NEU3 (55). The upregulation of NEU1 during the differentiation of monocytes into macrophages enhances the phagocytosis of these cells (56). However, in hepatocellular carcinoma, tumors with high NEU1 expression exhibited lower infiltration of B cells, CD8^+^ T cells, neutrophils, NK cells, pDCs, T helper cells, Th1 cells, and tumor infiltrating lymphocytes (TILs) compared to those with low NEU1 expression (57). In melanoma carcinoma, study suggests that high NEU1 expression is associated with lower levels of CD4^+^ T cells, B cells, and NK cells, and higher levels of Treg cells (39). The impact of NEU1 on cervical cancer immune cells, however, is unclear.

In this study, we provided insights into the causal effects between celecoxib target gene NEU1 inhibitor, specific immune cells, and cervical cancer. Our findings suggested that CD25 on CD45RA^+^ CD4 non-regulatory T cells might be involved in the association between the NEU1 inhibitor and cervical cancer. Moreover, our study provided genetic evidence that CD25 on CD45RA^+^ CD4 non-regulatory T cell might mediate the protective effect of the NEU1 inhibitor against cervical cancer. However, it is important to highlight that the mediation association observed might not be causal and needs to be further validated through experimental studies.

Currently, research on CD25 in CD45RA^+^ CD4 non-regulatory T cells is limited. CD45RA is a marker for naïve T cells. In contrast, CD25 (IL-2Rα) is elevated in activated T cells. Therefore, CD25 on CD45RA^+^ CD4 non-regulatory T cells may represent a type of non-regulatory T cell that's in an early activation phase or transitioning from resting to activation (58). The decrease in the proportion of this cell population in cervical cancer may be closely related to T cell exhaustion and immune escape. A study by Kanegane H et al.' suggested that CD25^+^ CD4^+^ T cells with the CD45RA^+^ phenotype might be cells activated by antigenic stimulation *in vivo* recently, while in transition into memory cells (58). An integrated immunological analysis of single-cell and bulk tissue transcriptomes demonstrated that in cervical cancer, the higher the degree of immune infiltration levels of resting memory CD4^+^ T cells (45RA^+^) and M0 macrophages, the better the clinical outcomes (59). Ou Y, et al. found that the loss of the CD45RA marker on activated naïve T cells is linked to worse clinical outcomes in patients. The loss of CD45RA marker hinders the immune system's ability to mount an effective response against tumor cells (9).

This study demonstrated that NEU1 inhibition is associated with increased CD25 expression, suggesting that NEU1 may regulate the IL-2R signaling pathway through glycosylation modification. Previous studies have shown that NEU1 can mediate the desialylation of various membrane receptors (such as TLR4, FcγR, EGFR, etc.), thereby affecting their signal transduction intensity (43). We speculate that in the immune microenvironment of cervical cancer, NEU1 inhibitor may block IL-2R desialylation, stabilize CD25 expression, enhance IL-2 signal, and promote T cell proliferation and anti-tumor effects. However, this mechanism requires further investigation and validation through experimental studies.

This study has several strengths. First, to our knowledge, the potential immune mechanisms underlying the association between celecoxib and cervical cancer have not been explored using the MR method. This study provided genetic evidence on the potential mechanism of the beneficial effect of an NEU1 inhibitor on cervical cancer through CD25 on CD45RA+ CD4+ non-regulatory T cells. Compared with observational studies, the MR analyses in this study can mitigate residual confounding and reverse causation. Furthermore, the two-sample MR design increases statistical power since GWAS summary data were extracted from at least two different datasets, instead of only one as used in one-sample MR.

Although this study combines pharmacological experiments, network pharmacology, and MR analyses to elucidate the potential mechanisms of the antitumor effects of celecoxib in cervical cancer, several limitations should be acknowledged. First, the genetic variants used as proxies for NEU1 inhibition reflect lifelong exposure, and the effect sizes of these variants may not accurately reflect the short-term impact of a pharmacological NEU1 inhibitor. Second, while we deliberately employed a nominal significance threshold (*P* < 0.05) in our multidimensional immune cell analysis to generate biological hypotheses and prevent the omission of potential signals resulting from excessive correction (22, 60)—a common practice in exploratory MR studies (22)—this approach inherently increases the risk of false-positive findings. To mitigate this, we restricted our interpretation on signals demonstrating directional consistency and biological plausibility. Additionally, the eQTL and GWAS data utilized were sourced from public databases. Although these datasets primarily originate from distinct research projects, the possibility of sample overlap cannot be entirely excluded, which could theoretically introduce potential bias in MR estimates. Notably, all instrumental variables exhibited *F*-statistics greater than 10, indicating robust instrument strength that helps mitigate weak instrument bias. However, given these potential limitations, future studies based on completely independent samples are needed to further validate the robustness of our findings.

Furthermore, due to practical limitations in experimental conditions and the scope of the current study, additional mechanistic experiments—including NEU1 overexpression or knockout in cervical cancer cell lines and *in vivo* validation using xenograft models—were not performed. These experiments would provide further functional evidence for the role of NEU1 in mediating the effects of celecoxib and will be an important focus of our future studies. In addition, comprehensive molecular validation of COX-2 (PTGS2) and NEU1 expression at the mRNA and protein levels under different treatment conditions was not conducted experimentally. To partially address this limitation, we analyzed the expression patterns of NEU1 and PTGS2 in cervical cancer using multiple independent transcriptomic datasets, including integrated data from The Cancer Genome Atlas and the Genotype-Tissue Expression (GTEx) project, as well as three GEO cohorts (GSE14404, GSE30155, and GSE63514). These analyses consistently showed that NEU1 was upregulated in cervical cancer tissues, whereas PTGS2 exhibited variable expression patterns across datasets (Supplementary Figure S1). Collectively, although further experimental validation is warranted, the current findings provide genetic and bioinformatic evidence supporting the potential involvement of NEU1 in the antitumor effects mediaed by celecoxib.

In conclusion, this study supports the association between the NEU1 inhibitor, which is genetically predicted to target celecoxib, and immune cells in cervical cancer. Specifically, CD25 on CD45RA+ CD4 non-regulatory T cells mediated the protective effect of NEU1 inhibitor on cervical cancer. These findings provide genetic evidence elucidating the mechanism by which the NEU1 inhibitor reduces cervical cancer risk and may inform future mechanistic and clinical research.

## Data Availability

The original contributions presented in the study are included in the article/Supplementary material, further inquiries can be directed to the corresponding author.
