# Supplementary figures and images for: Exploring the celecoxib–cervical cancer relationship using in vitro, network pharmacology, and Mendelian randomization approaches
Source: Front Med (Lausanne). 2026 May 19;13:1777235. doi: 10.3389/fmed.2026.1777235 (PMC13226506; doi:10.3389/fmed.2026.1777235)

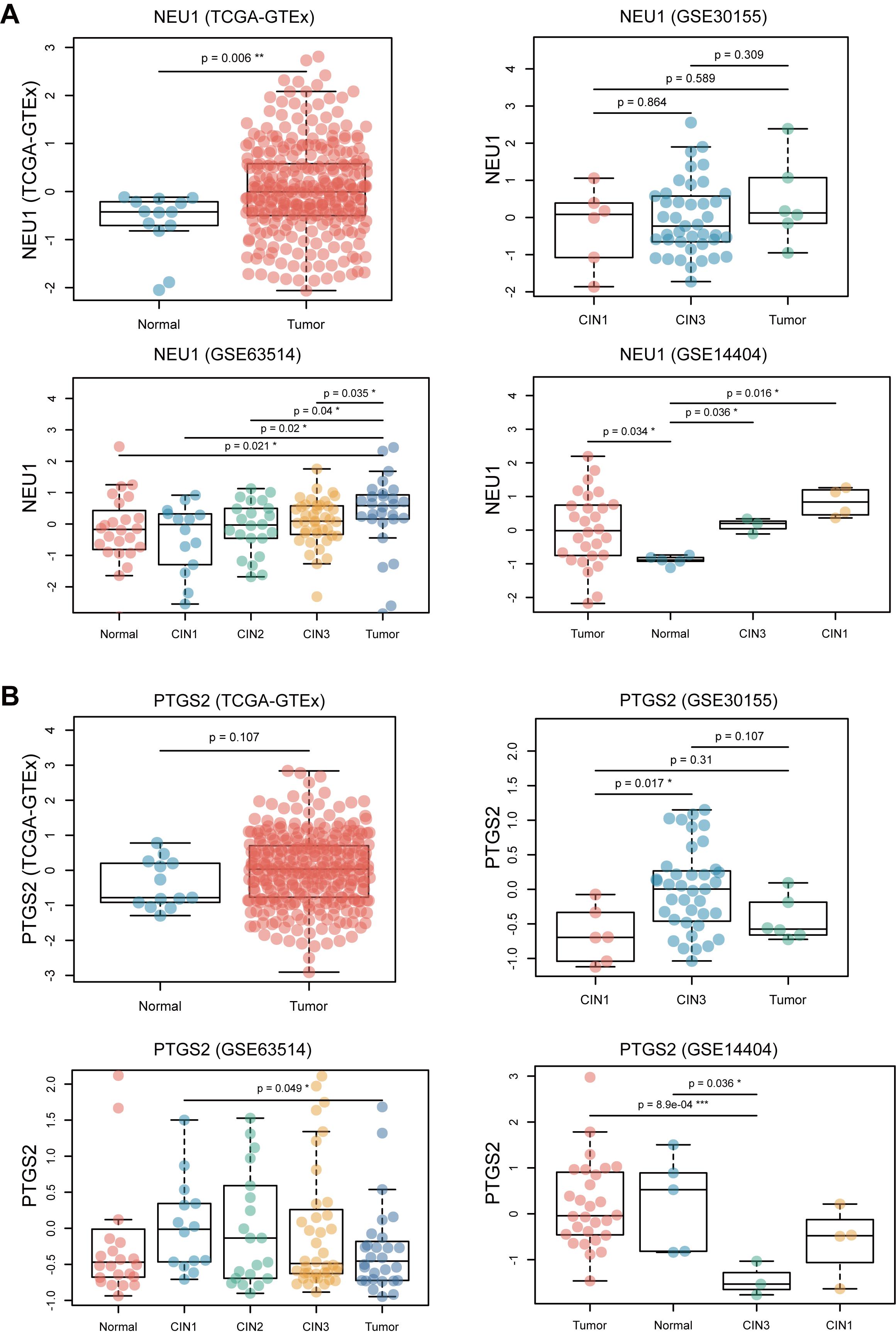

Supplement: Supplementary file 7 [file Image_1.jpeg]
